# Extraction of phenolic compounds and flavonoids from tomato residues using high-pressure-temperature advanced method

**DOI:** 10.3389/fchem.2026.1765834

**Published:** 2026-01-22

**Authors:** Nidal Zrikam, Amine Ezzariai, Youssef El Kharrassi, Abdelaziz Nilahyane, Sabrine Zouiete, Kamal Aberkani, Layla El Gueddari, Loubna El Fels, Yedir Ouhdouch, Lamfeddal Kouisni, Mohamed Hafidi, Adil Mazar

**Affiliations:** 1 African Sustainable Agriculture Research Institute (ASARI), University Mohammed VI Polytechnic (UM6P), Laâyoune, Morocco; 2 Laboratory of Microbial Biotechnologies, Agrosciences and Environment (BioMAgE), Labeled Research Unit-CNRST N°4, Faculty of Sciences Semlalia, Cadi Ayyad University, Marrakesh, Morocco; 3 Faculté Poly-Disciplinaire de Nador, University Mohammed Premier, Oujda, Morocco

**Keywords:** anti-elastase, antioxidant, circular bioeconomy, green extraction, high-pressure-temperature extraction, phenolic compounds, tomato residues, valorization

## Abstract

Tomato residues in the Agadir region constitute a large and under-exploited source of biomass, rich in bioactive molecules such as phenolic and flavonoid compounds. This study highlights, for the first time, the use of high-pressure-temperature reactor as an innovative and advanced extraction technology to recover phenolic acids and flavonoids from stems and leaves of tomato waste. A multivariate optimization approach was designed to assess the effect of temperature, pressure, extraction time, and solvent ratio on the extraction efficiency to determine the optimal conditions. The performance of this method was compared to conventional and emerging techniques (Soxhlet, maceration, and ultrasound-assisted extraction), while the biological activities of the extracts were evaluated via their antioxidant and enzymatic properties. The results showed a maximum extraction yield of 31.2% for stems and 53.9% for leaves under moderate conditions (25 °C, 180 min, 10 bar, 30% ethanol). The highest levels of phenolic compounds (1240.89 mg GA/g extract) and flavonoids (59.32 mg QE/g extract) were obtained at 160 °C and 10 bars, with ethanol concentrations between 70% and 100%. Pareto analysis identified temperature and solvent polarity as the key variables influencing extraction efficiency. The optimal extracts demonstrated strong antioxidant activity (up to 85% DPPH inhibition and 261.8 mg trolox/g extract in the FRAP test) as well as significant anti-elastase potential (>90% inhibition), highlighting their potential for cosmetic and nutraceutical applications. Compared to conventional and advanced techniques already available on the market, Parr reactor extraction offers superior yield, selectivity, and process efficiency. This study validates its role as an environmentally friendly and scalable alternative for the recovery of tomato processing waste within a circular bioeconomy.

## 1 Introduction

Agriculture is one of the industrial sectors that generates the most waste. Certain agricultural industries produce around 1.3 billion tons of waste per year (Kour et al., 2023; Sadh et al., 2018). In the agro-industry, biomass undergoes various stages of processing to recover various agro-industrial by-products. The tomato (*Solanum lycopersicum*), a member of the Solanaceae family, is one of the most widely consumed vegetables in the world (Padmanabhan et al., 2016). Fruits and vegetables are among the main sources of industrial by-products (Gustavsson et al., 2011). Losses and waste associated with these commodities, corresponding to end products that are not utilized or are diverted to other uses, can reach up to 50% of production, particularly during processing and post-harvest stages (De Brito Nogueira et al., 2020). Waste generated from tomato cultivation is attracting growing interest worldwide as an under-exploited source of bioactive compounds, such as polyphenols and carotenoids (Branthôme, 2020; FAO, 2021). Tomato by-products are rich in organic matter (68%–74%) and contain valuable plant nutrients, including nitrogen, carbon, and calcium, which promote plant growth (Tabrika et al., 2021). Their lignocellulosic composition typically consists of 20.9% cellulose, 12% hemicellulose, and 6.9% lignin (Bascón-Villegas et al., 2020).

The components of tomato residues are known for their high content of bioactive molecules such as polyphenols and flavonoids, which have antibacterial, antifungal, and antiviral properties and may play a role in defending plants against pathogens (Arab et al., 2019). Flavonoids are among the main compounds in tomatoes, contributing to their aroma and colour. These substances have anti-inflammatory activity in the intestine and have been associated with the prevention of gastric cancer (Nishiumi, 2011). These high-value bioactive molecules have applications in many sectors. Their recovery and extraction represent a promising avenue for a new emerging scientific field at the interface of food science and technology and the valorization of bio-residues (Gomes et al., 2022).

The extraction efficiency depends on many factors such as particle size, solvent type, ratio of biomass to solvent, extraction time and method, as well as temperature. Common extraction methods include mechanical agitation, ultrasound-assisted extraction, Soxhlet extraction, maceration, and supercritical fluid extraction (Ochoa-Villarreal, 2012). Studies indicate that tomato leaves and stems contain 39–70.8 mg GA/g total phenolic content and 18.1–32.3 mg EC/g total flavonoids (Añibarro-Ortega et al., 2020). Tomato crop waste (stems and leaves) was extracted using ultrasound (40 kHz) at 70 °C with methanol as a solvent mixed with hydrochloric acid, as well as by maceration for 20 min under the same conditions. Certain conditions made it possible to obtain significant quantities of phenolic acids and flavonoids (Aires et al., 2016). Microwave-assisted extraction has also proven effective in recovering flavonoids and polyphenols while producing antioxidant ingredients (Pinela et al., 2016). Bioactive molecules were identified using a Soxhlet apparatus with various solvents, including ethanol, 2-methyltetrahydrofuran (2-MTHF), and ethyl acetate. GC-MS analysis revealed the presence of various molecules, such as polyphenols, aldehydes, and alcohols (Drescher et al., 2025). The yield and selectivity of conventional extraction methods generally depend on the polarity of the solvent and can degrade sensitive compounds when heat is applied. In contrast, ultrasound-assisted extraction improves the recovery of phenolic compounds and flavonoids (Bekavac et al., 2025). In addition, phenolic compounds and flavonoids from tomato agro-industrial waste are generally extracted using ethanol-water mixtures with orbital shaking at high temperatures for prolonged periods of time (Solaberrieta et al., 2022). However, ultrasonic extraction achieves comparable yields in significantly shorter times (Gomes et al., 2022). In the field of research and development, some extraction technologies such as microwave-assisted extraction, pressurized liquid extraction, and supercritical fluid extraction are being investigated to recover bioactive compounds from agricultural by-products. The efficiency of these methods depends on mass transfer and the desorption of target molecules from the matrix to the solvent, which reduces extraction times and solvent consumption (Dzah and Dzigbor, 2023). High-temperature extraction contributes to the mechanism of increasing phenolic acids due to their insolubility resulting from the breakdown of the bond between lignin and phenolic acids (Maillard and Berset, 1995). Extraction under high hydrostatic pressure is considered as one of the most simplest and effective extraction techniques (FDA, 2014). Extraction using high pressure allows for the inactivation and inhibition of enzymes and the preservation of food quality (Yamamoto, 2017). It has been shown that this type of extraction induces changes in biomass by promoting the bioavailability and stability of bioactive molecules (Cilla et al., 2012). Pressure and temperature have a synergistic effect on molecules by forcing the solvent to penetrate the matrix and increasing the diffusivity of the solvent and their solubility. At high temperatures, the tension and viscosity of the solvent decrease completely, leading to mass transfer and ensuring more efficient extraction than at low temperatures (Kamali et al., 2016; Machado et al., 2015). High-temperature, high-pressure extraction has become a widely studied technique due to its ability to provide an integrated system that facilitates multiple simultaneous reactions while ensuring high extraction yields and superior extract quality (Li J. et al., 2022). The HPTR, one of the most used tools for this type of extraction, was used to study the effects of extraction time and temperature on the recovery of phenolic compounds and flavonoids from olive pomace. The results showed excellent total content of polyphenol (45.2 mg CAE/g DP) and flavonoid (15.3 mg CE/g DP) (Aliakbarian et al., 2011). Other studies have shown that the HPTR can efficiently extract monomeric phenolic compounds while simultaneously producing synthesis gas and valuable phenolic monomers (Curmi et al., 2022). In addition, this reactor allows the recovery of valuable compounds with anti-inflammatory, anti-allergic, and antimicrobial properties (Szabo et al., 2018).

Although it offers significant advantages, the High-temperature-Pressure (HPTR) reactor has not yet been explored for developing a validated method for extracting and recovering phenolic acids and flavonoids from tomato agro-industrial biomass residues. The current paper aims to explore, the extraction of bioactive molecules from tomato plant residues using this reactor. The influence of key parameters (temperature, duration, pressure, and solvent concentration) is examined, and the results are compared with those obtained using conventional extraction methods (cold maceration and Soxhlet extraction) and a more advanced method (ultrasound-assisted extraction). Following extraction, the phytochemical profiles of polyphenols and flavonoids were determined, and antioxidant and enzymatic activities were evaluated.

## 2 Materials and methods

### 2.1 Preparation of the raw material

Agro-industrial tomato residues were collected from an agro-industrial facility designated for tomato production in Agadir. The residues were sorted into leaves and stems, then dried in an oven at 60 °C, crushed using a stem crusher, and sieved to 250–500 μm before being stored at room temperature until analysis.

### 2.2 Physicochemical characterization of the raw material

The pH and electronic conductivity (EC) values were measured on an aqueous extract of the plant materiel at room temperature (1 g/10 mL of distilled water) in accordance with standard AFNOR NF T90-008. To determine the dry matter and moisture content, the oven-drying method was used: a porcelain capsule, previously reduced to a constant mass, was placed in an oven for 24 h at a temperature of T = 105 °C. After drying, the capsules were cooled in a desiccator and weighed. To determine the volatile matter and ash content, the capsule used to determine the moisture content was heated in a muffle furnace at 550 °C for 5 h, then calcined, repeating the cycle of cooling, and weighing the capsule mass. Nitrogen is determined by the Kjeldahl method (Kjeldahl, 1883). In this procedure, the nitrogenous organic matter in the plant sample is mineralized by a mixture of salicylic acid and concentrated sulfuric acid under the effect of heat and in the presence of a catalytic mixture. In this way, nitrogen is converted to ammonia. The distillate is collected in boric acid. It is then titrated with a 0.1 N HCl solution. The protein content is calculated by multiplying the nitrogen content, determined by the Kjeldahl method, by a conversion factor (N) representing the average nitrogen content of proteins.

### 2.3 Biochemical characterization of the raw material

#### 2.3.1 Total sugars content

The total sugar content is measured using the method described by (DuBois et al., 1956). This method involves heating concentrated mineral acids to dehydrate hexoses and pentoses, followed by cyclization to form furfural derivatives such as 5-hydroxymethylfurfural, which react with phenol. If sugars are present, a yellow complex is formed. The concentration of this complex is measured by spectrophotometry at a wavelength of 480 nm.

#### 2.3.2 Extraction and measurement of photosynthetic pigments

A sample of approximately 50 mg from leaves was dissolved in 4 mL of 80% acetone (3 mL for grinding and 1 mL for rinsing). After 2 h in the dark, the optical densities (OD) were read at 480 nm, 645 nm, and 663 nm (Arnon, 1949). The following formulas, based on Beer-Lambert’s law, can be used to calculate pigment concentrations:

$$\text{Chl }a\;\left(\text{mg }g\text{ of dry }{\text{weight}}^{-1}\right)=\left[12.7\left(\text{OD }663\right)-2.69\left(\text{OD }645\right)\times V\;/\;1000\times W\right]$$

$$\text{Chl }b\;\left(\text{mg }g\;d.w.^{-1}\right)=\left[22.9\left(\text{OD }645\right)-4.68\;\left(\text{OD }663\right)\times V\;/\;1000\times W\right]$$

Carotenoids (mg g of dry matter^−1^) = Acar/Em × 100, where V represents the volume of the aliquot and W represents the weight of the tissue. Acar = DO 480 + 0.114 (DO 663) - 0.638 (DO 645) and Em = 2500.

### 2.4 Extraction optimization using the HPTR

Extraction experiments were conducted in a HPTR (Series 5000 Multiple Reactor System) designed to provide an integrated system for performing multiple reactions simultaneously and applying high-throughput experimentation principles to reactions conducted at high temperatures and pressures. The experiments were conducted by varying the following extraction parameters: temperatures (25, 50, 70, and 160 °C), times (30 min, 60 min, and 180 min), pressure (5 and 10 bar), and ethanol concentration (30%, 70%, and 100%) (Table 1). For each test, 2 g of material was suspended in the solvent medium (40 mL) and then subjected to the different extraction conditions. The extracts were then centrifuged for 10 min at 5,000 rpm and the supernatants were concentrated using a rotary vacuum evaporator. All experiments were performed in triplicate.

**TABLE 1:** Variables used in optimizing extraction by the HPTR.

Temperature (°C)	Extraction time (min)	Solvent concentration (ethanol v/v)	Pressure (bar)
25	30	30	5
50	60	70	10
70	180	100	​
160	​	​	​

### 2.5 Extraction using conventional methods

#### 2.5.1 Cold maceration extraction

5 g of material (leaves and stems) were placed in a 100% ethanol solution (70:30 v: v) and stirred at a temperature of 4 °C for 24 h. The solid material was collected by filtration and the solvent was evaporated under vacuum.

#### 2.5.2 Soxhlet extraction

The extraction process was carried out using a Soxhlet apparatus with 5 g of sample powder and 100 mL of an ethanol and water solution (70:30 v: v) for 6 h at a temperature of 60 °C. The solvent was then evaporated using a rotary evaporator.

#### 2.5.3 Ultrasound-assisted extraction

Ultrasound-assisted extraction of tomato residues (leaves and stems) was performed using a sonicator (QSonica Q500, power 500 W, 20 kHz, 25 mm probe, maximum amplitude of 120 μm) under control of time, temperature, amplitude, and pulse. 5 g of material (leaves and stems) were treated in ethanol solvent (70:30 v: v). The mixture was then sonicated at a specified amplitude for a specified extraction time. The extract was centrifuged at 3000 *g* for 10 min and concentrated in a rotary vacuum evaporator.

### 2.6 Determination of extraction yield

The extraction yield (%) was determined by calculating the ratio of the dry extract mass to the dry matter mass used: extraction yield (%) = 100 × extract weight/solid weight.

### 2.7 Phytochemical screening

#### 2.7.1 Determination of total polyphenol content

The polyphenol concentration in the aqueous extracts was determined using the Folin-Ciocalteu method (Vlase et al., 2014), with glycolic acid as the standard. To 0.25 mL of each extract, 1 mL of Folin-Ciocalteu reagent (10%) and 750 μL of sodium carbonate solution (1%) were added. After incubation for 2 h at room temperature, the absorbance was measured at 760 nm. The analyses were performed in triplicate and the results were expressed in mg of gallic acid equivalent (GA)/gram of extract.

#### 2.7.2 Determination of total flavonoid content

The flavonoid content was determined using the method described by Herald et al. (2012). This method consists of adding 250 μL of an ethanol solution (2% AlCl3) to 250 μL of each extract (1 mg/mL). The mixture is incubated for 30 min at room temperature and the absorbance is measured at 415 nm. The flavonoid content is expressed in mg of quercetin equivalent (Q)/gram of extract.

### 2.8 Evaluation of biological activities

#### 2.8.1 Antioxidant activities

The (2,2-diphenyl-1-picrylhydrazyl-hydrate) free radical scavenging activity (DPPH) was evaluated according to the method described by Molyneux (2004). A methanolic solution of DPPH was prepared by dissolving 4 mg of this product in 100 mL of ethanol with stirring for 30 min. Next, 1.5 mL of the DPPH solution was added to 500 μL of extract at a concentration of (1 mg/mL). The tubes were shaken for a few seconds and then incubated for 30 min in the dark. The absorbance was recorded using a spectrophotometer at a wavelength of 517 nm, using methanol as a blank. Ascorbic acid was used as a control. The antioxidant activity related to the DPPH radical scavenging effect is expressed as a percentage of inhibition (PI) using the following formula:

$$\text{Inhibition }\left(\%\right)=\left(\text{Abs control }517-\text{Abs extract }517/\text{ Abs control }517\right)\;*100$$

The ferric reducing antioxidant power (FRAP) test was performed by preparing the FRAP reagent (acetate buffer, TPTZ, and FeCl3⋅6H2O) and mixing 0.3 μL of extract with 2.7 mL of FRAP reagent, incubating for 10 min at 37 °C, and measuring the absorbance at 593 nm. The results are expressed in mg trolox/g extract (Nenadis et al., 2004).

#### 2.8.2 Enzymatic activity (anti-elastase)

A stock solution of porcine pancreatic elastase (3.33 mg/mL) was prepared in 0.2 mM Tris-HCl buffer (pH 8). The substrate, N-methoxy-succinyl-Ala-Ala-Pro-Val-pNA (0.43 mM), was dissolved in Tris-HCl buffer. The test consisted of pre-incubating 50 μL of the sample solution, Tris-HCl buffer, and enzyme for 15 min. Next, 50 μL of substrate was added to reach a final volume of 200 μL, and the reaction mixtures were incubated at 37 °C for 20 min. The absorbance was immediately measured at 405 nm using a microplate reader. Kojic acid was used as a positive control (Li S. et al., 2022). The percentage of elastase inhibition (%) was determined using the following formula:

$$\text{Inhibition }\left(\%\right)=\left(\text{Ac}-\text{As }/\text{ Ac}\right)\times 100$$

where, As: corrected absorbance of samples (A Sample + Enzyme – A Sample). Ac: absorbance of controls (A buffer + enzyme).

## 3 Results and discussions

### 3.1 Results of physicochemical and biochemical characterization

The Table 2 shows the physicochemical properties of tomato waste (leaves and stems) collected in the Agadir region. The results indicate significant physicochemical differences between the leaf and stem matrices. The pH of the leaves (6.10 ± 0.07) was significantly less acidic than that of the stems (5.65 ± 0.07). Additionally, the elevated electrical conductivity observed in the leaves (590.57 ± 278.90 µS/cm) suggests a substantially higher concentration of ionic solutes, such as mineral salts. The analysis demonstrate that the leaves have a high-water content (85%), as well as significant levels of nitrogen (3.961%), proteins (24%), and total sugars content (17.011 mg/g MF). The leaves from Agadir also had high levels of photosynthetic pigments, particularly chlorophyll a, with an average of 16.881 ± 4.08. These results could be attributed to a lack of fertilization (Perezespinosa, 2005), which could explain the variations in nitrogen, sugar, and protein content. As for tomato stems, the analysis revealed that they are richer in protein and nitrogen and have a very high-water content (90%).

**TABLE 2:** Physicochemical and biochemical characterization of parameters of tomato waste.

Parameters	Leaves	Stems
pH	6.10 ± 0.07	5.65 ± 0.07
Conductivity (µs/cm)	590.57 ± 278.90	369.37 ± 88.64
Moisture (%)	85.82 ± 1.02	90.04 ± 0.95
Dry matter (%)	14.18 ± 1.02	9.96 ± 0.95
Volatile matter (%)	81.60 ± 1.14	81.17 ± 2.13
Ash content (%)	18.40 ± 1.14	18.40 ± 2.3
Nitrogen (%)	3.97 ± 0.32	3 ± 0.22
Proteins (%)	24.76 ± 1.98	18.77 ± 1.38
Total sugars content (mg/g MF)	17.01 ± 10.47	8.29 ± 0.07
Chlorophyll a (µg/mL)	16.89 ± 4.08	-
Chlorophyll b (µg/mL)	3.35 ± 1.99	-
Carotenoids (µg/mL)	4.82 ± 2.19	-

### 3.2 Evaluation of stem extracts obtained using HPTR

#### 3.2.1 Analysis of extraction yield, total polyphenol content (TPC), and total flavonoid content (TFC)

The optimization of tomato stem waste extraction using a HPTR was carried out by testing 216 operating conditions. The extraction yields obtained during these experiments ranged from 3.02% ± 2.3% to 31.2% ± 4.7%. The maximum yield (31.2% ± 4.7%) was achieved under the following conditions: a temperature of 25 °C, an extraction time of 180 min, an ethanol concentration of 30%, and a pressure of 10 bar. Conversely, the lowest yields were observed at temperatures between 25 °C and 50 °C under a pressure of 5 bar. The results indicate a positive correlation between extraction time and yield prolonged contact between the solvent and the biomass results in a higher extractable mass. By comparison, conventional extraction methods, with a duration of 60 min, yield much lower yields, ranging from 8% to 14% (Añibarro-Ortega et al., 2020; Perea-Domínguez et al., 2018). Under optimal conditions, extraction techniques such as microwaves and ultrasound have achieved yields of 11% and 13.2% respectively, with ultrasound extraction proving slightly more effective (Hassan et al., 2014). This difference could be explained by improved mass transfer during ultrasonic extraction, which is likely to release higher molecular weight compounds. Another study comparing the extraction yield from different parts of tomato waste found that the extraction yields for the stems and leaves were ≤20%, even though this waste is rich in bioactive molecules that were the subject of this study for the formulation of advanced soil cover biofilms for hydroponic tomato crops in the context of the circular economy (Panagiotopoulou et al., 2022).

The results of the phytochemical screening show TPCs ranging from 8.76 ± 31.05 to 289.36 ± 31.05 mg GA/gE. The maximum average TPC (289.36 ± 31.05 mg GA/gE) recorded is the result of the following experimental conditions (temperature = 70 °C, extraction time = 30 min, ethanol concentration = 70%, pressure = 10 bars). By varying the extraction temperature, the polyphenol content remains high compared to other conditions even if the extraction time is short and the pressure is 5 bar. By varying ethanol concentration, there is no significant difference between concentrations of 70% and 100% in terms of TPC, however a concentration of 30% slightly reduces the polyphenol content under the conditions mentioned above. Temperature has been shown to be an important factor in extracting bioactive compounds (Cheynier et al., 2013). A previous study used tomato waste as biomass to develop a better extraction technique to produce extracts with improved polyphenol levels based on physical parameters, extraction time, solvent, and liquid-solid ratio. As a result, Soxhlet and ultrasound extraction yielded higher amounts of TPC: 178.20 mg GA/gE for ultrasound and 73.79 mg GA/gE for Soxhlet, but ultrasound was complete in only 5 min. Microwave extraction yielded a TPC of 146.57 mg GA/gE and was fast, completing the extraction in 20 min, but reducing the phenol concentration by about 30% compared to the other two methods (López-Téllez et al., 2024).

The maximum flavonoid content reached 47.90 ± 10.80 mg QE/gE, obtained under the following conditions (temperature = 70 °C, extraction time = 30 min, ethanol concentration = 100%, pressure = 10 bars). The accumulation of flavonoids in tomatoes is a dynamic process, positively influenced by light but inversely affected by darkness (Wilkens et al., 1996). Beyond these immediate environmental factors, the final content is highly dependent on the cultivar, as established by several studies (Stewart et al., 2000; Willcox et al., 2003). The impact of climate is also notable, with plants from warmer regions consistently showing higher flavonoid concentrations than those from more temperate climates (Stewart et al., 2000). Previous studies have shown the presence of 6 mg/g of biomass of flavonoids in extracts obtained from tomato waste (leaf part) using deep eutectic solvents by extraction under mechanical agitation at a temperature of 70 °C for 2 h (Wawoczny et al., 2025).

#### 3.2.2 Influence of extraction parameters on extraction yield

**FIGURE 1:**
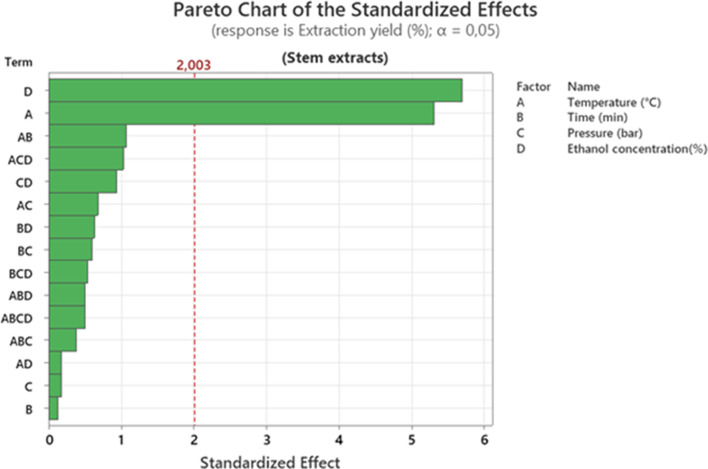
Pareto chart representation of the effects of extraction factors on the extraction yield of stem extracts.

Analysis of the Pareto chart (Figure 1) of normalized effects shows that ethanol concentration is the most decisive variable in optimizing extraction yield. Its effect, which is significantly above the statistical significance threshold (α = 0.05), indicates that increasing or modulating this parameter directly influences the efficiency of the process. It has been suggested that the effectiveness of using polar solvent as an extraction agent interacts with high temperatures in ASE extractions at 165 °C (Efthymiopoulos et al., 2018). Temperature also appears to be a major factor, confirming that the solubilization and diffusion of bioactive compounds are strongly linked to the thermal conditions applied. Work carried out using other extraction methods, such as microwaves, has highlighted the role of increased temperature in breaking down cell walls, thereby promoting the release of target compounds (Carpentieri et al., 2021). The temperature range has been adjusted between 50 °C and 100 °C, which can have an impact on extraction yield (Bhadange et al., 2024). Apparently, most modern extraction techniques use high temperatures to increase extraction yield. It has been observed that the extraction time and pressure parameters do not have a statistically significant effect on the response variable, suggesting that their contribution is negligible in the intervals tested. Furthermore, the influence of certain interactions, although detectable, remains below the critical threshold, highlighting the predominance of the main effects in this process.

#### 3.2.3 Influence of extraction parameters on polyphenol content (TPC)

**FIGURE 2:**
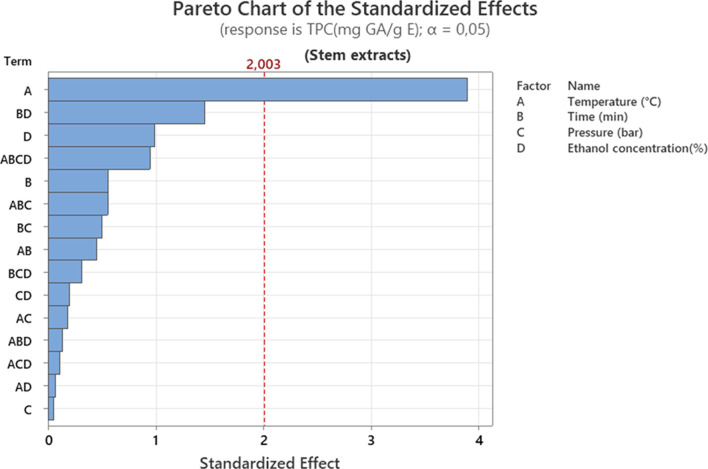
Pareto chart representation of the effect of different extraction factors on total polyphenol content (TPC) of stem extracts.

The Figure 2 shows that temperature (A) is the most influential parameter, well above the significance threshold. The AB interaction (temperature × time) also plays a secondary role. These results indicate that polyphenol extraction is highly dependent on temperature control, which is consistent with the heat-sensitive nature of these molecules and their increased release at high temperatures. One study found that solvent extraction under pressure increases yield at temperatures above 100 °C. Raising the temperature to between 180 °C and 200 °C results in a significant increase in TPC, as well as enhanced antioxidant activity in the extracts (Antony and Farid, 2022).

#### 3.2.4 Influence of extraction parameters on flavonoids content (TFC)

**FIGURE 3:**
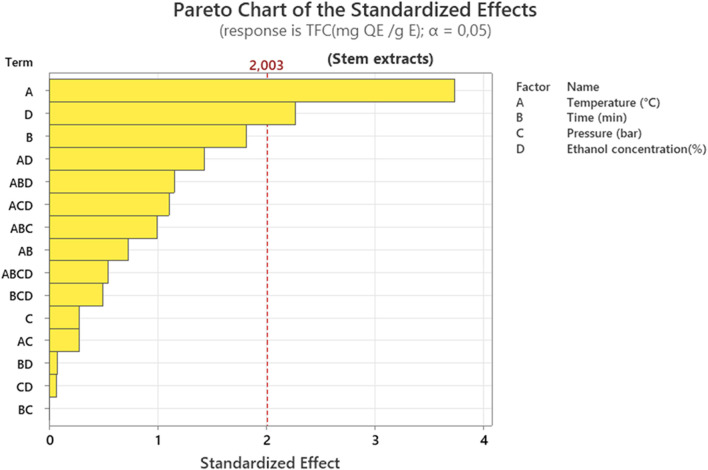
Pareto chart representation of the effect of different extraction factors on total flavonoid content (TFC) of stem extracts.

This Pareto chart presented in Figure 3 reveals that temperature is the predominant factor controlling flavonoid content (TFC), with its normalized effect of 2.003 being the only one to clearly exceed the significance threshold (α = 0.05). Ethanol concentration (factor D) has a very marked effect. Its interaction with temperature (AD) is also significant, as are secondary interactions such as AB and BD. This suggests that optimizing the solvent, particularly the proportion of ethanol, is crucial for flavonoid extraction. On the other hand, extraction time, pressure, and ethanol concentration have secondary or even negligible influences. This hierarchy indicates that process optimization should focus on controlling temperature, which probably affects the diffusion kinetics and solubility of the target compounds, potentially reducing the other less influential parameters for more efficient extraction.

### 3.3 Evaluation of leaves extracts obtained using HPTR

#### 3.3.1 Analysis of extraction yield, total polyphenol content (TPC), and total flavonoid content (TFC)

The phytochemical screening results obtained for the leaf extracts show that under conditions of temperature 25 °C, extraction time of 60 min, pressure 10 bars, and a solvent concentration of 70% ethanol, the overall extraction yield is 53.95%. The results obtained show that the operational conditions strongly influence the composition of the extracts. Indeed, an extraction performed at a high temperature of 160 °C, with a reduced duration of 30 min, a pressure of 10 bars, and an ethanol concentration of 30% yielded a TPC = 1240.89 ± 53.62 mg GA/gE. A previous study reports that some heat treatments increase TPC, perhaps because they cause the release of compounds contained in the vacuoles of fruits and vegetables, in addition to cell degradation and the denaturation of oxidative and hydrolytic enzymes capable of degrading polyphenols (Fuentes et al., 2013). The change from a polyphenol content of 11.09 ± 5.493 mg GA/gE at a temperature of 25 °C to a value of 124.89 ± 53.62 mg GA/gE at a temperature of 160 °C shows that temperature has a positive linear effect on polyphenols. This hypothesis was confirmed by Helmi et al. (2025), who worked on the extraction from the foliar part of tomato across a temperature range from 25 °C up to 80 °C, and they found that TPC increases with rising temperature, reaching 30 mg GA/g DM under conditions of 78.5 °C and a duration of 29 min. The improvement in polyphenol recovery also depends on the extraction solvent used; for this reason, using ethanol for extraction from tomato leaves is more effective than using water alone.

Furthermore, the use of a 100% ethanol concentration at 25 °C and 10 bars achieved a TFC of 59.32 mg QE/gE, which highlighted the importance of simultaneously optimizing temperature, time, and solvent polarity to target specific compounds of interest and maximize the added value of the extracts. A comparative study between two tomato varieties revealed very different TFCs, following the same extraction procedure: 7.8 ± 0.15 mg RE/gE for the first variety and 35.5 ± 1.64 mg RE/g E for the second variety, showing that the flavonoid concentration depends on the type of cultivar of the plant in question (Omotoyinbo et al., 2020).

#### 3.3.2 Influence of extraction parameters on extraction yield

**FIGURE 4:**
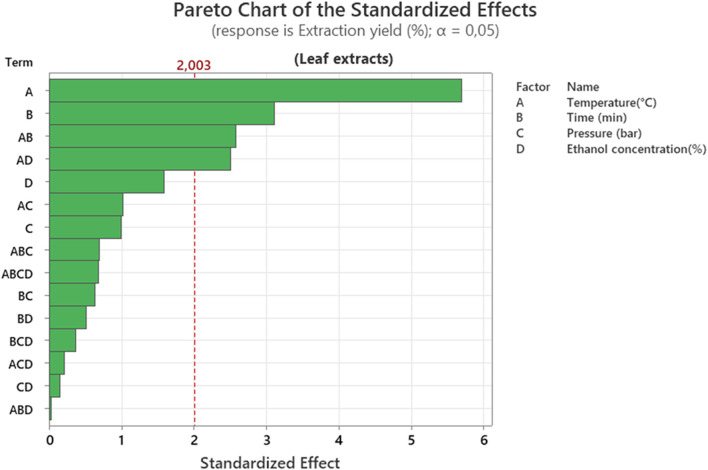
Pareto chart representation of the effect of different extraction factors on extraction yield of leaf extracts.

The Pareto diagram (Figure 4) shows that the dominant factor is temperature (A), followed by extraction time (B). Significant interactions are also observed, such as AB (temperature × time) and AD (temperature × ethanol concentration). This indicates that the yield is primarily influenced by temperature and time, confirming that thermal elevation facilitates the solubilization of bioactive compounds. When compared with stem extracts, which also show that extraction yield is impacted by temperature as well as ethanol concentration. Overall, these results confirm that optimizing extraction yield mainly relies on controlling solvent concentration and temperature, followed by extraction duration, while other parameters can be adjusted secondarily without significantly affecting process efficiency.

The increase in temperature disrupts the matrix interactions between bioactive molecules and the samples, which are caused by hydrogen bonds, van der Waals forces, and dipole interactions. This facilitates deeper solvent penetration into the sample matrix and increases extraction yield (Mustafa and Turner, 2011). Another study also confirmed that temperature affects extraction yield by increasing both solvent solubility and the mass transfer rate within the plant matrix studied (Pangestuti et al., 2021). As for extraction time, it remains one of the factors that need to be optimized in each extraction to help minimize cost and energy consumption (Spigno et al., 2007).

#### 3.3.3 Influence of extraction parameters on polyphenol content (TPC)

**FIGURE 5:**
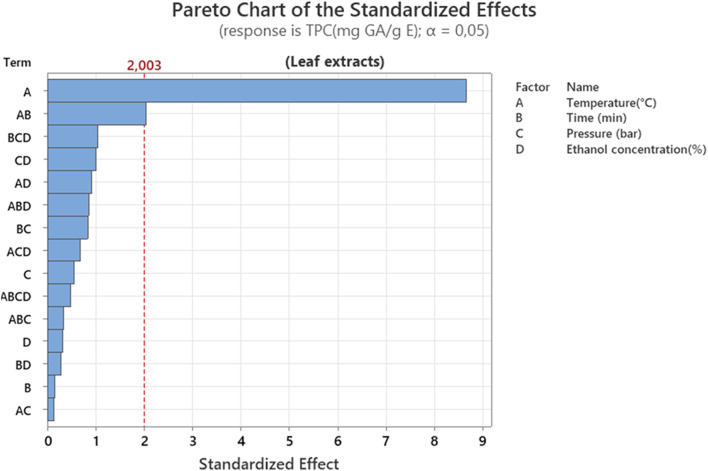
Pareto chart representation of the effect of different extraction factors on total polyphenol content (TPC) of leaf extracts.

Observation of the Figure 5 reveals that temperature (A) is the most influential parameter, far exceeding the significance threshold. The AB interaction (temperature × time) also plays a secondary role. These results indicate that the extraction of polyphenols is highly dependent on thermal control, which is consistent with the heat-sensitive nature of these molecules and their increased release at high temperatures. One study established that pressurized solvent extraction increases yield at temperatures above 100 °C. A temperature increase to a range of 180 °C–200 °C leads to a significant increase in TPC, as well as enhanced antioxidant activities of the extracts (Antony and Farid, 2022).

#### 3.3.4 Influence of extraction parameters on flavonoids content (TFC)

**FIGURE 6:**
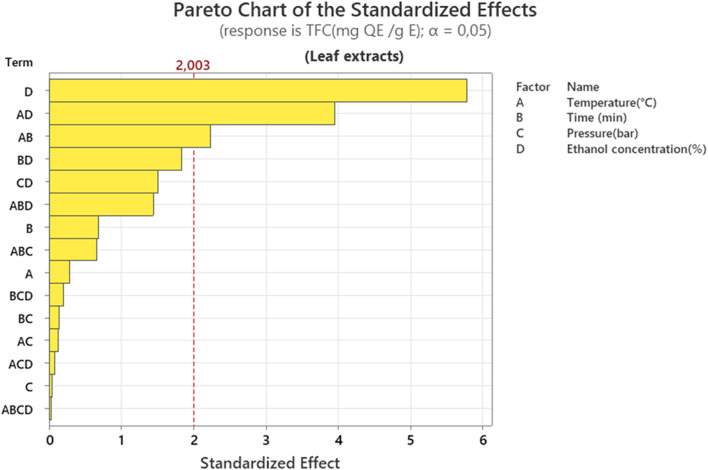
Pareto chart representation of the effect of different extraction factors on total flavonoid content (TFC) of leaf extracts.

The ethanol concentration (D) has the most significant effect, followed by its interaction with temperature (AD). The importance of interactions AB and BD is also observed (Figure 6). This demonstrates that the proportion of solvent is the key parameter for the extraction of flavonoids, as their solubility is influenced by the polarity of the ethanol and water mixture. Furthermore, temperature promotes flavonoid extraction at temperatures between 25 °C and 50 °C. Above this temperature, the flavonoid concentration decreased slightly. This can be explained by the fact that higher temperatures are not favorable for flavonoid extraction due to the evaporation of the ethanol solvent (Wei and Yang, 2014). In addition, the extraction solvent influences the extraction of secondary metabolites; water is not a preferred solvent for flavonoids, however, the ethanol/water mixture was preferable for flavonoids, with the four concentrations of 50%, 60%, 70%, and 80% were tested in an experiment, the flavonoid extraction yield reached a maximum of 9,263 mg/g with an ethanol concentration of 70%. Beyond this threshold, the yield increases with concentration, but decreases sharply beyond that point. This peak efficiency can be explained by the optimal solubility of flavonoids in 70% ethanol. This leads to the conclusion that a higher concentration probably reduces the extraction of water-soluble compounds such as flavonoids (Bi et al., 2011).

### 3.4 Selection of optimum conditions

There are few publications dealing with the qualitative determination of bioactive compounds in extracts from tomato leaf or stem waste using HPTR. Studies conducted by the HPTR on other biomasses show that under high pressure and high temperature conditions, maximum TPC (23.8 mg GA/gE) and TFC (15.5 mg QE/gE) contents were obtained at a temperature of 150 °C after 240 min under a nitrogen atmosphere (Ben Hamissa et al., 2012). Based on the previous results highlighting the effects of extraction factors, a selection of optimal operating conditions with an excellent phytochemical profile and poor conditions with a poor phytochemical profile was made by cross-referencing the mass extraction yield of the HPTR with quantitative analyses of polyphenols and flavonoids (Tables 3, 4). These selected conditions will be subject to further evaluation of their antioxidant and enzymatic activity. The objective of this step is to confirm the performance of extraction by the HPTR, which is a method applied here for the first time in this context.

**TABLE 3:** Selected conditions for stem extracts.

Extracts		Temperature (°C)	Time (min)	Pressure (bar)	Ethanol concentration (%)
Low conditions	T1	25	30	5	30
	T4	25	30	10	30
	T3	25	30	5	100
	T5	25	30	5	70
	T12	25	60	10	100
	T17	25	180	5	70
Optimum conditions	T71	160	180	5	70
	T72	160	180	10	100
	T59	160	30	5	70
	T56	160	30	10	70
	T61	160	60	5	30
	T64	160	60	10	30

**TABLE 4:** Selected conditions for leaf extracts.

Extracts		Temperature (°C)	Time (min)	Pressure (bar)	Ethanol concentration (%)
Low conditions	F3	25	30	5	100
	F4	25	30	10	30
	F21	50	30	5	100
	F24	50	30	10	100
	F27	50	60	5	100
	F42	70	30	10	100
Optimum conditions	F59	160	30	5	70
	F56	160	30	10	70
	F61	160	60	5	30
	F62	160	60	10	70
	F68	160	180	10	70
	F69	160	180	5	100

### 3.5 Antioxidant activity of the extracts

**FIGURE 7:**
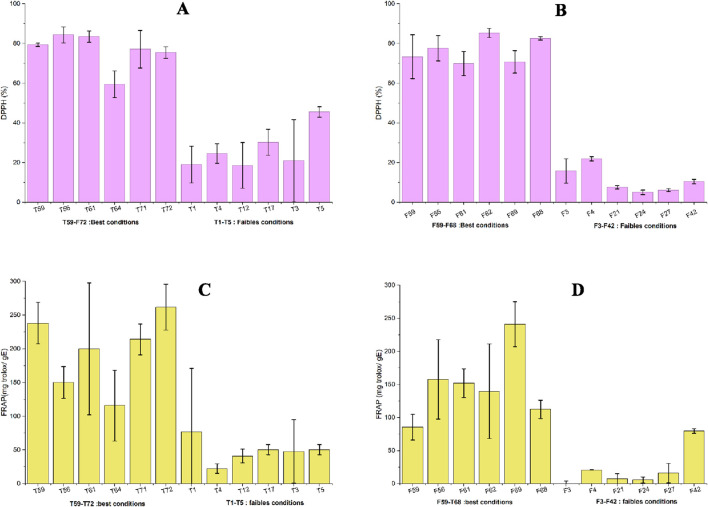
Antioxidant activity results for selected extracts; **(A)** DPPH test (%) for stem extracts, **(B)** DPPH test (%) for leaf extracts, **(C)** FRAP test (mg trolox/gE) for stem extracts, **(D)** FRAP test (mg trolox/gE) for leaf extracts.

The extracts obtained from tomato stems (T59–T72, Figure 7A) and leaves (F59–F68, Figure 7B) under optimal conditions exhibited high antioxidant activity, reaching 70%–85% DPPH inhibition, indicating an efficient extraction of phenolic compounds. In contrast, extracts obtained under optimal conditions, with low phytochemical profiles in terms of TPC and TFC (stems T1–T5 and leaves F3–F42), showed markedly reduced activity (<40%), highlighting the major influence of extraction parameters on the recovery of antioxidant molecules. TPCs act as effective hydrogen donors to the DPPH radical due to their specific chemical structure. Their antioxidant capacity depends mainly on the number and position of hydroxyl groups on their aromatic rings. As shown in Figure 7, the high levels of TPCs and TFCs in the selected extracts correlate positively with DPPH activity. TPCs thus appear to be efficient hydrogen donors to the DPPH radical owing to their ideal chemical structure. This antioxidant activity is attributed to the presence of phenolic compounds in the extracts, whose efficiency depends on their structure, particularly the number and position of hydroxyl groups on the aromatic rings (Balasundram et al., 2006; Turkmen et al., 2007).

The antioxidant activity measured by the FRAP assay was very high in the selected stem extracts (T59–T72, Figure 7C), reaching a maximum value of 261.814 mg trolox/g extract in T72, obtained at 160 °C, 180 min, 10 bar, and using pure ethanol (100%). All major selected extraction conditions were performed at high temperatures (160 °C). Conversely, extracts obtained under mild conditions (T1–T5) exhibited lower FRAP activity, ranging from 76.41 to 22.55 mg trolox/g E.

For the leaf extracts, Figure 7D shows similar FRAP trends to the stem extracts. The maximum value among extracts F3–F42 was close to 250 mg trolox/g E, whereas extracts from mild conditions (F3–F42) showed FRAP values below 100 mg trolox/g E. It is noteworthy that extracts F3, F4, F21, F24, and F27, obtained at 25 °C, exhibited very low FRAP activity (<25 mg trolox/g E). In contrast, extract F42, obtained under similar conditions but at a higher temperature (70 °C), showed a marked increase in FRAP value.

This clear increase in FRAP antioxidant activity with varying extraction conditions suggests that temperature plays a key role in enhancing the antioxidant potential of tomato waste extracts. Higher temperatures can promote compound diffusion but may also reduce extraction selectivity and affect thermolabile compounds (Mustafa and Turner, 2011). Comparatively, previous studies reported FRAP antioxidant activity of tomato fruits reaching 23.6 ± 6.3 mg TE/g (Ganugi et al., 2023). Other studies found a value of 23.73 µmol/g for FRAP activity in tomato agro-industrial waste extracts obtained by non-conventional extraction techniques (Chada et al., 2022).

The results presented in Figure 7 suggest that high TPC and TFC contents in the selected extracts are positively correlated with antioxidant activities as measured by both DPPH and FRAP assays. These bioactive compounds can inhibit oxidative processes induced by free radicals (Minarti et al., 2024). Overall, these findings demonstrate that a moderate increase in temperature can enhance the diffusion and recovery of antioxidant compounds, leading to the identification of an optimal extraction point using HPTR, and confirming the biological value of the extracts for potential agro-food or cosmetic applications.

### 3.6 Anti-elastase biological potential

**FIGURE 8:**
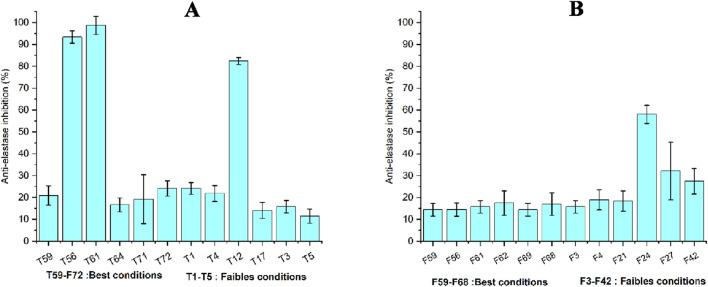
Results of anti-elastase activity of stem **(A)** and leaf **(B)** extracts using HPTR.

The results of the percentage inhibition of anti-elastase activity show a wide fluctuation between stem extracts obtained under optimal and suboptimal conditions. Figure 8A shows that, the excellent extracts T56 and T61 recorded the highest inhibition rates, close to 100%, while T12 exhibited around 80% inhibition of elastase activity. In a previous study on tomatoes, following extraction by maceration using ethanol (70%) as a solvent, the mean inhibition rate of elastase activity was 19.73% ± 0.44%. This result correlated positively with their antioxidant activity (Henderson et al., 2020).

The anti-elastase activity results of leaf extracts (Figure 8B) showed lower inhibition rates compared to stem extracts. Elastase is a proteolytic enzyme that hydrolyzes the peptide bonds of elastin a key structural protein responsible for skin elasticity. Excessive elastase activity in the dermis leads to the degradation of elastin, contributing to skin aging. Therefore, elastase inhibition represents an effective cosmetic strategy to prevent loss of elasticity. Compounds capable of inhibiting this enzyme are thus developed as active ingredients for anti-aging formulations (Ambarwati et al., 2019). It can be observed that the stem extracts contain more compounds than the leaf extracts that may influence elastase inhibition. The ability of our extracts to inhibit this enzyme may not be explained solely by their phytochemical profile; however, in some stem extracts, TPC and TFC appear to contribute to elastase inhibitory activity. Most natural antioxidants are phenolic compounds that can reduce oxidant levels and inhibit enzymes such as elastase. It has been demonstrated that the polyphenol content of plants helps to combat free radicals that trigger the skin-aging process (Wittenauer et al., 2015).

These selected extracts obtained under both optimal and suboptimal conditions showed moderate to high inhibition values. Considering that they were derived from agro-industrial waste, it is noteworthy that this anti-elastase activity exhibits remarkable potential, especially in the fruit and seed parts of tomato plants.

### 3.7 Comparative evaluation with the extraction methods

**FIGURE 9:**
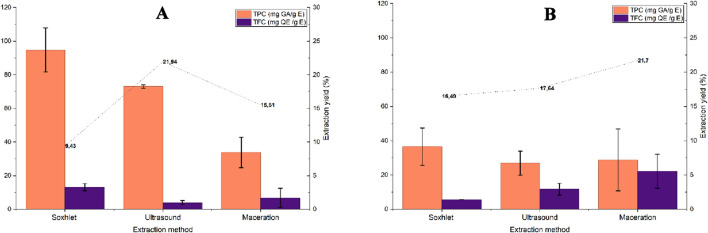
Results of total polyphenol and flavonoid content obtained based on the extraction yield of tomato waste; stem part **(A)** and leaf part **(B)** using the three conventional methods.

The results of phytochemical analysis of tomato extracts obtained using the two conventional methods and one advanced method are shown in Figure 9. For stem extracts (Figure 9A), extraction using Soxhlet showed a very low average extraction yield (9.3%), with very low TPC levels close to 95 mg GA/gE and low TFC levels with an average of 13.15 ± 2.10 mg QE/gE. Extraction by ultrasound and maceration showed a slight increase in extraction yield, 17.64% and 21.70% respectively. In terms of bioactive molecules, ultrasound showed an average TPC of 73.03 ± 1 mg GA/gE and TFC of 3.95 ± 2.10 mg QE/gE, while maceration showed an average TPC of 33.76 ± 9.04 mg GA/gE and TFC of 6.85 ± 5.61 mg QE/gE.

For leaf extracts, the graph shown in (Figure 9B) indicates a low extraction yield (16.49%) for Soxhlet extraction compared to the other two techniques. However, Soxhlet extraction yields the highest TPCs, with an average of 36.56 ± 10.93 mg GA/gE, and a low TFC of 5.62 ± 0.04 mg QE/gE. Moving on to the ultrasound method, we see that it gives results slightly similar to Soxhlet, with average TPCs of 26.96 ± 7 mg GA/gE, and TFCs of 11.72 ± 3.49 mg QE/gE. Finally, extraction using cold maceration gives TPC values close to 30 mg GA/gE and TFC values close to 20 mg QE/gE, slightly higher than those obtained by the other methods, with a maximum extraction yield of 21.70%. Extraction using the Soxhlet apparatus favors stable total phenols, while maceration promotes the preservation and diffusion of sensitive flavonoids. Ultrasound remains in second place between the Soxhlet apparatus and maceration, as a balanced alternative between yield, speed, and selectivity.

**FIGURE 10:**
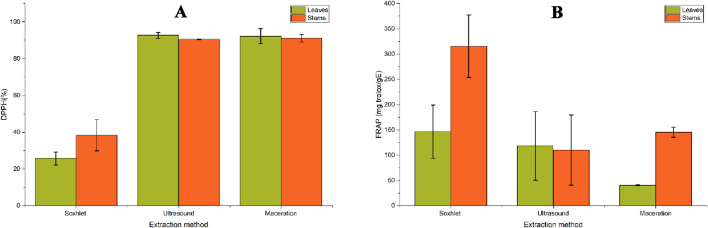
Antioxidant activity (%) results using the DPPH assay **(A)** and FRAP assay (mg trolox/gE) **(B)** of extracts obtained from tomato waste using three different extraction techniques.

The comparative evaluation of different extraction techniques (Soxhlet, ultrasound, and maceration) highlights marked contrasts in terms of overall yield and richness in bioactive compounds (TPC and TFC). The results show that the Soxhlet method leads to the highest total polyphenol content, close to 100 mg GA/gE, followed by ultrasound-assisted extraction, while maceration remains the least effective for this category of metabolites. These observations can be explained mainly by the thermal and exhaustive nature of the Soxhlet process, in which continuous solvent reflux promotes the disintegration of cell walls and the complete solubilization of phenolic compounds trapped in the lignocellulosic matrix. However, this same prolonged heating can lead to partial thermal degradation of certain flavonoids and other sensitive metabolites, which explains the relatively moderate TFC contents observed. Moving on to the antioxidant activity results (Figure 10), Soxhlet extraction of stem extracts shows DPPH antioxidant activity of less than 25%. However, the percentage of DPPH inhibition rises to 89% for the last two extraction methods using extracts from stems and leaves. Antioxidant activity, as measured by the DPPH test, was strongly correlated with TPC and TFC. The low DPPH antioxidant activity capacity could be linked to the low polyphenol and flavonoid content in these extracts, as the very long maceration process generates a low yield at room temperature, and its long extraction time may lead to the risk of fermentation and degradation of the target molecules.

The FRAP test results show that extracts obtained using the Soxhlet method have the highest values, reaching approximately 315.44 mg trolox/g of extract for stems and 170 mg trolox/g for leaves. Stems consistently show higher FRAP activity than leaves, regardless of the method used. In contrast, ultrasound-assisted extraction generated intermediate values (approximately 120–140 mg trolox/g), indicating moderate efficiency. This method, considered gentler and more respectful of the chemical integrity of metabolites, allows for effective cavitation but is sometimes limited by the resistance of the plant matrix, particularly in more lignified stems. Maceration leads to the lowest values (40 mg trolox/g for leaves and 100 mg trolox/g for stems), which can be explained by the absence of mechanical agitation and heat, thus reducing the diffusion and solubilization of the active compounds.

**FIGURE 11:**
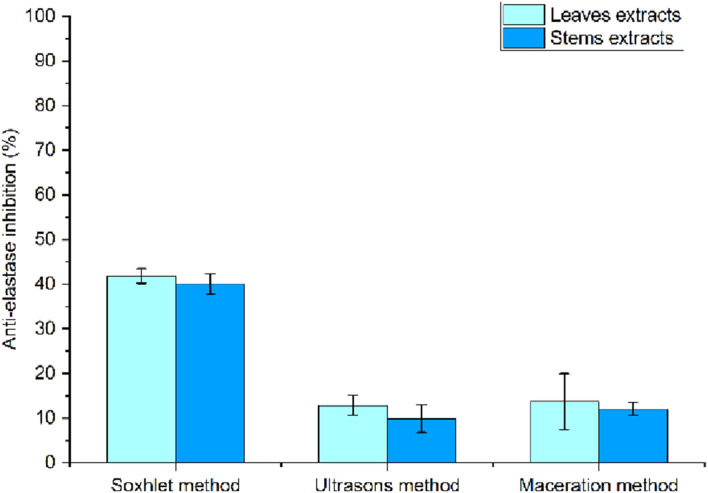
Anti-elastase activity of tomato waste extracts using conventional methods.

The other aspect of the evaluation of biological activities concerns anti-elastase enzyme activity, which demonstrates the anti-aging potential of our extracts using other methods (Figure 11). The graphs show high inhibitory activity from Soxhlet, with an average value of 41.80% ± 1.66% for leaves and 40.09% ± 2.30% for stems. This enzymatic activity then drops for both types of stem and leaf extracts obtained using ultrasound and maceration, with inhibition varying between 9% and 13%. The superior efficiency of the Soxhlet method can be explained by the extraction conditions (temperature and extraction time), which allow for better release of molecules with anti-elastase activity. In comparison, ultrasound and maceration techniques, although more environmentally friendly and less aggressive, are less effective at targeting this specific enzymatic activity.

TPCs were higher in tomato samples extracted using solid-liquid extraction than in other samples extracted using Soxhlet extraction. The polyphenol content also depends on another factor, namely, the polarity of the solvent, with semi-polar and polar solvents being favorable for phenolic compounds (Brglez Mojzer et al., 2016; Drescher et al., 2025). In a study on tomatoes, maceration extraction yielded polyphenol concentrations ranging from 3.2 to 3.8 mg GA/g (Peralta et al., 2020). Antioxidant activity, as assessed by the DPPH test, was strongly correlated with TPC and TFC. This relationship confirms the major contribution of phenolic compounds to the elimination of free radicals and the modulation of oxidative stress (Marcu Spinu et al., 2025). The variation in flavonoids in this study was due to the dominant influence of temperature and solvent polarity, while time and pressure had secondary effects. Statistical analysis using Pareto charts revealed that temperature is the most important factor in the extraction of flavonoids from tomato stems, while ethanol concentration and its interaction with temperature play a more important role for leaf extracts. In stems, increasing the temperature has a positive effect on flavonoid extraction yield, while in leaves, solvent concentration is the predominant factor. Furthermore, it has been shown that certain flavonoids are capable of neutralizing free radicals. Their mechanism of action is based on the transfer or donation of electrons, as well as the inhibition of the activity of enzymes or lipids involved in the production of these same radicals (Samsonowicz et al., 2015).

For anti-elastase activity, Soxhlet extraction showed higher inhibition than ultrasound and maceration. When combined with the mild conditions of HPTR, these extracts exhibit antioxidant and anti-aging activities similar to those obtained using traditional methods, demonstrating that HPTR allows for the adjustment of secondary metabolite selectivity (TPC and TFC) and maximization of the activities (DPPH/FRAP/anti-elastase) of tomato waste. Furthermore, the best extraction conditions selected from the HPTR extracts showed phytochemical profiles with high TPC and TFC contents. These extracts were positively correlated with their biological activities, which showed DPPH activity ranging from 70% to 85% and FRAP activity reaching a maximum value of 261,814 mg trolox/gE. In addition, anti-elastase inhibition rates reached notable values between 20% and 98%.

These results are consistent with previous studies, indicating that high-pressure and high-temperature extraction can lead to an increase in chemical and biochemical reactions in cells through both desirable and undesirable changes, which influences its antioxidant capacities (Oey et al., 2008). It has been shown that certain insoluble molecules contained in plants, as well as lignin, degrade at high temperatures, leading to an increase in phenolic acids and extraction yields (Maillard and Berset, 1995). A study has shown that the innovative high-pressure extraction technique preserves the valuable active ingredients in plants. It thus provides access to the wealth of essential micronutrients and bioactive phytochemicals contained in fruits and vegetables (Wang et al., 2016).

Overall, the use of HPTR in this study as an extraction technique leads to results superior to those obtained under traditional conditions when the parameters are optimized. It also allows comparable efficiency to be achieved under less intense operating conditions. The results indicate that the range studied, pressure between 5 and 10 bar had a negligible influence on the extraction yield and bioactive compound recovery. Furthermore, no significant interaction between pressure and the other studied factors was observed. This suggests that increasing pressure under these conditions does not enhance extraction efficiency, which may be advantageous from a process scalability and economic perspective, as lower pressure systems are simpler and less energy intensive. This study showed that the most influential parameters are temperature and solvent polarity. These two factors are decisive for extraction yield and the preservation of the bioactivity of the extracts. These results represent a promising strategy for the sustainable recovery of agro-industrial tomato waste.

## 4 Conclusion

This work demonstrates the feasibility of extraction assisted by the HPTR, which significantly improved the extraction yield of bioactive compounds. Under optimal conditions (160 °C, 10 bar, 70%–100% ethanol), the stem and leaf extracts showed a high total phenolic content (up to 1240.9 mg EAG/g extract) and a high total flavonoid content (59.3 mg EQ/g extract). This optimization identified temperature and solvent polarity as the dominant parameters. The evaluation of biological activities confirmed the high functional potential of the extracts, with DPPH radical inhibition reaching 70%–85%, FRAP activity up to 261.8 mg trolox/g extract, and elastase inhibition close to 100%. This process establishes a new approach to valorizing tomato residues and contributes to the development of sustainable pathways for biomass valorization within the framework of a circular bioeconomy.

## Data Availability

The original contributions presented in the study are included in the article/supplementary material, further inquiries can be directed to the corresponding author.

## References

[B1] Aires A. Carvalho R. Saavedra M. J. (2016). Valorization of solid wastes from chestnut industry processing: extraction and optimization of polyphenols, tannins and ellagitannins and its potential for adhesives, cosmetic and pharmaceutical industry. Waste Manag. 48, 457–464. 10.1016/j.wasman.2015.11.019 26626811

[B2] Aliakbarian B. Casazza A. A. Perego P. (2011). Valorization of olive oil solid waste using high pressure–high temperature reactor. Food Chem. 128 (3), 704–710. 10.1016/j.foodchem.2011.03.092

[B3] Ambarwati N. S. S. Elya B. Desmiaty Y. (2019). Anti-elastase activity of methanolic and ethyl acetate extract from Garcinia latissima Miq. J. Phys. Conf. Ser. 1402 (5), 055079. 10.1088/1742-6596/1402/5/055079

[B4] Añibarro-Ortega M. Pinela J. Ćirić A. Martins V. Rocha F. Soković M. D. (2020). Valorisation of table tomato crop by-products: phenolic profiles and *in vitro* antioxidant and antimicrobial activities. Food Bioprod. Process. 124, 307–319. 10.1016/j.fbp.2020.09.006

[B5] Antony A. Farid M. (2022). Effect of temperatures on polyphenols during extraction. Appl. Sci. 12 (4), 2107. 10.3390/app12042107

[B6] Arab M. Bahramian B. Schindeler A. Valtchev P. Dehghani F. McConchie R. (2019). Extraction of phytochemicals from tomato leaf waste using subcritical carbon dioxide. Innovative Food Sci. and Emerg. Technol. 57, 102204. 10.1016/j.ifset.2019.102204

[B7] Arnon D. I. (1949). Copper enzymes in isolated chloroplasts polyphenoloxidase in beta vulgaris. Plant Physiol. 24 (1), 1–15. 10.1104/pp.24.1.1 16654194 PMC437905

[B8] Balasundram N. Sundram K. Samman S. (2006). Phenolic compounds in plants and agri-industrial by-products: antioxidant activity, occurrence, and potential uses. Food Chem. 99 (1), 191–203. 10.1016/j.foodchem.2005.07.042

[B9] Bascón-Villegas I. Espinosa E. Sánchez R. Tarrés Q. Pérez-Rodríguez F. Rodríguez A. (2020). Horticultural plant residues as new source for lignocellulose nanofibers isolation: application on the recycling paperboard process. Molecules 25 (14), 3275. 10.3390/molecules25143275 32708406 PMC7397013

[B10] Bekavac N. Radović M. Jurinjak Tusek A. Cvjetko Bubalo M. Zelić B. Šalić A. (2025). Smart design of deep eutectic solvent-based aqueous two-phase systems for efficient lipase extraction. Elsevier BV. 10.2139/ssrn.5256092

[B11] Ben Hamissa A. M. Seffen M. Aliakbarian B. Casazza A. A. Perego P. Converti A. (2012). Phenolics extraction from Agave americana (L.) leaves using high-temperature, high-pressure reactor. Food Bioprod. Process. 90 (1), 17–21. 10.1016/j.fbp.2010.11.008

[B12] Bhadange Y. A. Carpenter J. Saharan V. K. (2024). A comprehensive review on advanced extraction techniques for retrieving bioactive components from natural sources. ACS Omega 9 (29), 31274–31297. 10.1021/acsomega.4c02718 39072073 PMC11270575

[B13] Bi J. Yang Q. Sun J. Chen J. Zhang J. (2011). Study on ultrasonic extraction technology and oxidation resistance of total flavonoids from peanut hull. Food Sci. Technol. Res. 17 (3), 187–198. 10.3136/fstr.17.187

[B14] Branthôme F.-X. (2020). Global trade and consumption of tomato products in 2020.

[B15] Brglez Mojzer E. Knez Hrnčič M. Škerget M. Knez Ž. Bren U. (2016). Polyphenols: extraction methods, antioxidative action, bioavailability and anticarcinogenic effects. Molecules 21 (7), 901. 10.3390/molecules21070901 27409600 PMC6273793

[B16] Carpentieri S. Soltanipour F. Ferrari G. Pataro G. Donsì F. (2021). Emerging green techniques for the extraction of antioxidants from agri-food By-Products as promising ingredients for the food industry. Antioxidants 10 (9), 1417. 10.3390/antiox10091417 34573049 PMC8471374

[B17] Chada P. S. N. Santos P. H. Rodrigues L. G. G. Goulart G. A. S. Azevedo Dos Santos J. D. Maraschin M. (2022). Non-conventional techniques for the extraction of antioxidant compounds and lycopene from industrial tomato pomace (Solanum lycopersicum L.) using spouted bed drying as a pre-treatment. Food Chem. X 13, 100237. 10.1016/j.fochx.2022.100237 35498978 PMC9040000

[B18] Cheynier V. Comte G. Davies K. M. Lattanzio V. Martens S. (2013). Plant phenolics: recent advances on their biosynthesis, genetics, and ecophysiology. Plant Physiology Biochem. 72, 1–20. 10.1016/j.plaphy.2013.05.009 23774057

[B19] Cilla A. Alegría A. De Ancos B. Sánchez-Moreno C. Cano M. P. Plaza L. (2012). Bioaccessibility of tocopherols, carotenoids, and ascorbic acid from Milk- and soy-based fruit beverages: influence of food matrix and processing. J. Agric. Food Chem. 60 (29), 7282–7290. 10.1021/jf301165r 22738607

[B20] Curmi H. Chirat C. Roubaud A. Peyrot M. Haarlemmer G. Lachenal D. (2022). Extraction of phenolic compounds from sulfur-free black liquor thanks to hydrothermal treatment before the production of syngas for biofuels. J. Supercrit. Fluids 181, 105489. 10.1016/j.supflu.2021.105489

[B21] De Brito Nogueira T. B. Da Silva T. P. M. De Araújo Luiz D. De Andrade C. J. De Andrade L. M. Ferreira M. S. L. (2020). Fruits and vegetable-processing waste: a case study in two markets at Rio de Janeiro, RJ, Brazil. Environ. Sci. Pollut. Res. 27 (15), 18530–18540. 10.1007/s11356-020-08244-y 32193738

[B22] Drescher A. Schwingshackl L. Kienberger M. (2025). Identification of molecules from tomato plant residues using sustainable green chemicals. Biomass Convers. Biorefinery 15 (9), 14387–14398. 10.1007/s13399-024-06165-1

[B23] DuBois M. Gilles K. A. Hamilton J. K. Rebers P. A. Smith F. (1956). Colorimetric method for determination of sugars and related substances. Anal. Chem. 28 (3), 350–356. 10.1021/ac60111a017

[B24] Dzah C. S. Dzigbor A. (2023). Ultrasound assisted extraction: a relook at solvent to material ratio, its effects on process efficiency and how it can be exploited for different uses. J. Food Process Eng. 46 (7), e14339. 10.1111/jfpe.14339

[B25] Efthymiopoulos I. Hellier P. Ladommatos N. Russo-Profili A. Eveleigh A. Aliev A. (2018). Influence of solvent selection and extraction temperature on yield and composition of lipids extracted from spent coffee grounds. Industrial Crops Prod. 119, 49–56. 10.1016/j.indcrop.2018.04.008

[B26] FAO (2021). The state of food and agriculture 2021, Making agrifood systems more resilient to shocks and stresses. FAO. 10.4060/cb4476en

[B27] FDA (2014). Safe practices for food processes-kinetics of microbial inactivation for 564 alternative food processing technologies.

[B28] Fuentes E. Fuentes F. Vilahur G. Badimon L. Palomo I. (2013). Mechanisms of chronic state of inflammation as mediators that link Obese adipose tissue and metabolic syndrome. Mediat. Inflamm. 2013, 1–11. 10.1155/2013/136584 23843680 PMC3697419

[B29] Ganugi P. Fiorini A. Tabaglio V. Capra F. Zengin G. Bonini P. (2023). The functional profile and antioxidant capacity of tomato fruits are modulated by the interaction between microbial biostimulants, soil properties, and soil nitrogen status. Antioxidants 12 (2), 520. 10.3390/antiox12020520 36830078 PMC9951999

[B30] Gomes F. D. S. Silva L. O. M. Beres C. Pagani M. M. Brígida A. I. S. Santiago M. C. P. D. A. (2022). Processing tomato waste as a potential bioactive compounds source: phenolic compounds, antioxidant capacity and bioacessibility studies. Ciência Rural. 52 (2), e20201070. 10.1590/0103-8478cr20201070

[B31] Gustavsson J. Cederberg C. Sonesson (2011). Global food losses and food waste: extent, causes and prevention. Available online at: worldveg.tind.io.

[B32] Hassan E.-M. Hassane M. M. Areif M. H. Al-Amrousi E. F. (2014). Utilization of Egyptian tomato waste as a potential source of natural antioxidants using solvents, microwave and ultrasound extraction methods. Am. J. Food Technol. 10 (1), 14–25. 10.3923/ajft.2015.14.25

[B33] Helmi L. Sunoqrot S. Hijazi A. Alayli M. Rajha H. N. Al Bakri M. (2025). Tomato leaves as a sustainable biosorbent for the effective removal of some organic dyes and lead from water. Front. Environ. Sci. 13, 1615815. 10.3389/fenvs.2025.1615815

[B34] Henderson A. H. Lister I. N. E. Fachrial E. Girsang E. (2020). Antioxidant and anti-elastase activity of ethanol extract of tomato (Solanum lycopersicum L.). Bul. Penelit. Tanam. Rempah Dan Obat 31 (2), 67. 10.21082/bullittro.v31n2.2020.67-74

[B35] Herald T. J. Gadgil P. Tilley M. (2012). High‐throughput micro plate assays for screening flavonoid content and DPPH‐scavenging activity in sorghum bran and flour. J. Sci. Food Agric. 92 (11), 2326–2331. 10.1002/jsfa.5633 22419130

[B36] Kamali H. Khodaverdi E. Hadizadeh F. Ghaziaskar S. H. (2016). Optimization of phenolic and flavonoid content and antioxidants capacity of pressurized liquid extraction from Dracocephalum kotschyi *via* circumscribed central composite. J. Supercrit. Fluids 107, 307–314. 10.1016/j.supflu.2015.09.028

[B37] Kjeldahl J. (1883). Neue Methode zur Bestimmung des Stickstoffs in organischen Körpern. Fresenius’ Z. für Anal. Chem. 22 (1), 366–382. 10.1007/bf01338151

[B38] Kour R. Singh S. Sharma H. B. Naik T. S. S. K. Shehata N. N P. (2023). Persistence and remote sensing of agri-food wastes in the environment: current state and perspectives. Chemosphere 317, 137822. 10.1016/j.chemosphere.2023.137822 36649897

[B39] Li J. Pettinato M. Campardelli R. De Marco I. Perego P. (2022). High-pressure technologies for the recovery of bioactive molecules from agro-industrial waste. Appl. Sci. 12 (7), 3642. 10.3390/app12073642

[B40] Li S. Wang R. Hu X. Li C. Wang L. (2022). Bio-affinity ultra-filtration combined with HPLC-ESI-qTOF-MS/MS for screening potential α-glucosidase inhibitors from cerasus humilis (bge.) sok. Leaf-tea and *in silico* analysis. Food Chem. 373, 131528. 10.1016/j.foodchem.2021.131528 34774376

[B41] López-Téllez J. M. Cañizares-Macías M. D. P. Mir A. Saurina J. Núñez O. (2024). Characterization of the polyphenolic profile in tomato (*Lycopersicon esculentum* P. Mill) peel and seeds by LC-HRMS/MS. J. Agric. Food Chem. 72 (28), 15680–15692. 10.1021/acs.jafc.4c02126 38973576 PMC11261606

[B42] Machado A. P. D. F. Pasquel-Reátegui J. L. Barbero G. F. Martínez J. (2015). Pressurized liquid extraction of bioactive compounds from blackberry (Rubus fruticosus L.) residues: a comparison with conventional methods. Food Res. Int. 77, 675–683. 10.1016/j.foodres.2014.12.042

[B43] Maillard M.-N. Berset C. (1995). Evolution of antioxidant activity during kilning: role of insoluble bound phenolic acids of barley and malt. J. Agric. Food Chem. 43 (7), 1789–1793. 10.1021/jf00055a008

[B44] Marcu Spinu S. Dragoi Cudalbeanu M. Major N. Goreta Ban S. Palčić I. Ortan A. (2025). Box–behnken design optimization of green extraction from tomato aerial parts and axillary shoots for enhanced recovery of rutin and complementary bioactive compounds. Antioxidants 14 (9), 1062. 10.3390/antiox14091062 41008968 PMC12466514

[B45] Minarti M. Ariani N. Megawati M. Hidayat A. Hendra M. Primahana G. (2024). Potential antioxidant activity methods DPPH, ABTS, FRAP, total phenol and total flavonoid levels of Macaranga hypoleuca (reichb. F. and zoll.) leaves extract and fractions. E3S Web Conf. 503, 07005. 10.1051/e3sconf/202450307005

[B46] Molyneux P. (2004). The use of the stable free radical diphenylpicryl-hydrazyl (DPPH) for estimating antioxidant activity. arXiv 26 (2).

[B47] Mustafa A. Turner C. (2011). Pressurized liquid extraction as a green approach in food and herbal plants extraction: a review. Anal. Chim. Acta 703 (1), 8–18. 10.1016/j.aca.2011.07.018 21843670

[B48] Nenadis N. Wang L.-F. Tsimidou M. Zhang H.-Y. (2004). Estimation of scavenging activity of phenolic compounds using the ABTS^•+^ assay. J. Agric. Food Chem. 52 (15), 4669–4674. 10.1021/jf0400056 15264898

[B49] Nishiumi S. Miyamoto S. Kawabata K. Ohnishi K. Mukai R. Murakami A. (2011). Dietary flavonoids as cancer-preventive and therapeutic biofactors. Front. Biosci. S3 (1), 1332–1362. 10.2741/229 21622274

[B50] Ochoa-Villarreal M. (2012). “Plant cell wall polymers: function, structure and biological activity of their derivatives,” in Polymerization (London, United Kingdom: InTech). 10.5772/46094

[B51] Oey I. Van Der Plancken I. Van Loey A. Hendrickx M. (2008). Does high pressure processing influence nutritional aspects of plant based food systems? Trends Food Sci. and Technol. 19 (6), 300–308. 10.1016/j.tifs.2007.09.002

[B52] Omotoyinbo O. V. Awojulu E. O. Sanni D. M. (2020). Phytochemical screening, antioxidant and tyrosinase inhibitory studies of methanol leaf extracts of two tomato varieties. Highlights Biosci., 20216. 10.36462/H.BioSci.20216

[B53] Padmanabhan P. Cheema A. Paliyath G. (2016). “Solanaceous fruits including tomato, eggplant, and peppers,” in Encyclopedia of food and health (Elsevier), 24–32. 10.1016/B978-0-12-384947-2.00696-6

[B54] Panagiotopoulou M. Papadaki S. Missirli T. Thanassoulia I. Krokida M. (2022). Exploring the valorisation potential of tomato cultivation By-Products in the frame of circular economy. Waste Biomass Valorization 13 (9), 3957–3972. 10.1007/s12649-022-01786-x

[B55] Pangestuti R. Shin K.-H. Kim S.-K. (2021). Anti-photoaging and potential skin health benefits of seaweeds. Mar. Drugs 19 (3), 172. 10.3390/md19030172 33809936 PMC8004118

[B56] Peralta M. I. Fuentes K. N. Canalis A. M. Soria E. A. (2020). Effect of cultivation method and processing on total polyphenols content and antioxidant capacity of tomatoes (Solanum lycopersicum). Nutr. Clínica Y Dietética Hosp. 40 (3). 10.12873/403peralta

[B57] Perea-Domínguez X. P. Hernández-Gastelum L. Z. Olivas-Olguin H. R. Espinosa-Alonso L. G. Valdez-Morales M. Medina-Godoy S. (2018). Phenolic composition of tomato varieties and an industrial tomato by-product: free, conjugated and bound phenolics and antioxidant activity. J. Food Sci. Technol. 55 (9), 3453–3461. 10.1007/s13197-018-3269-9 30150804 PMC6098776

[B58] Perezespinosa A. Moral R. Moreno-Caselles J. Cortés A. Perez-Murcia M. D. Gómez I. (2005). Co phytoavailability for tomato in amended calcareous soils. Bioresour. Technol. 96 (6), 649–655. 10.1016/j.biortech.2004.07.002 15588767

[B59] Pinela J. Prieto M. A. Carvalho A. M. Barreiro M. F. Oliveira M. B. P. P. Barros L. (2016). Microwave-assisted extraction of phenolic acids and flavonoids and production of antioxidant ingredients from tomato: a nutraceutical-oriented optimization study. Sep. Purif. Technol. 164, 114–124. 10.1016/j.seppur.2016.03.030

[B60] Sadh P. K. Duhan S. Duhan J. S. (2018). Agro-industrial wastes and their utilization using solid state fermentation: a review. Bioresour. Bioprocess. 5 (1), 1. 10.1186/s40643-017-0187-z

[B61] Samsonowicz M. Kamińska I. Kalinowska M. Lewandowski W. (2015). Alkali metal salts of rutin – synthesis, spectroscopic (FT-IR, FT-Raman, UV–VIS), antioxidant and antimicrobial studies. Spectrochimica Acta Part A Mol. Biomol. Spectrosc. 151, 926–938. 10.1016/j.saa.2015.07.027 26184478

[B62] Solaberrieta I. Mellinas C. Jiménez A. Garrigós M. C. (2022). Recovery of antioxidants from tomato seed industrial wastes by microwave-assisted and ultrasound-assisted extraction. Foods Basel, Switz. 11 (19), 3068. 10.3390/foods11193068 36230144 PMC9562903

[B63] Spigno G. Tramelli L. De Faveri D. M. (2007). Effects of extraction time, temperature and solvent on concentration and antioxidant activity of grape marc phenolics. J. Food Eng. 81 (1), 200–208. 10.1016/j.jfoodeng.2006.10.021

[B64] Stewart M. Brown J. B. Donner A. McWhinney I. R. Oates J. Weston W. W. (2000). The impact of patient-centered care on outcomes. J. Fam. Pract. 49 (9), 796–804. 11032203

[B65] Szabo K. Cătoi A.-F. Vodnar D. C. (2018). Bioactive compounds extracted from tomato processing by-Products as a source of valuable nutrients. Plant Foods Hum. Nutr. 73 (4), 268–277. 10.1007/s11130-018-0691-0 30264237

[B66] Tabrika I. Mayad E. H. Furze J. N. Zaafrani M. Azim K. (2021). Optimization of tomato waste composting with integration of organic feedstock. Environ. Sci. Pollut. Res. 28 (45), 64140–64149. 10.1007/s11356-020-12303-9 33400108

[B67] Turkmen N. Velioglu Y. S. Sari F. Polat G. (2007). Effect of extraction conditions on measured total polyphenol contents and antioxidant and antibacterial activities of black tea. Molecules 12 (3), 484–496. 10.3390/12030484 17851405 PMC6149426

[B68] Vlase L. Benedec D. Hanganu D. Damian G. Csillag I. Sevastre B. (2014). Evaluation of antioxidant and antimicrobial activities and phenolic profile for Hyssopus officinalis, Ocimum basilicum and Teucrium chamaedrys. Molecules 19 (5), 5490–5507. 10.3390/molecules19055490 24786688 PMC6270679

[B69] Wang C.-Y. Huang H.-W. Hsu C.-P. Yang B. B. (2016). Recent advances in food processing using high hydrostatic pressure technology. Crit. Rev. Food Sci. Nutr. 56 (4), 527–540. 10.1080/10408398.2012.745479 25629307

[B70] Wawoczny A. Wilk J. Shyntum D. Shakibania S. Krukiewicz K. Gibas J. (2025). Valorization of waste tomato leaves with natural deep eutectic solvents. Food Chem. 472, 142884. 10.1016/j.foodchem.2025.142884 39826513

[B71] Wei M.-C. Yang Y.-C. (2014). Extraction characteristics and kinetic studies of oleanolic and ursolic acids from Hedyotis diffusa under ultrasound-assisted extraction conditions. Sep. Purif. Technol. 130, 182–192. 10.1016/j.seppur.2014.04.029

[B72] Wilkens R. T. Spoerke J. M. Stamp N. E. (1996). Differential responses of growth and two soluble phenolics of tomato to resource availability. Ecology 77 (1), 247–258. 10.2307/2265674

[B73] Willcox J. K. Catignani G. L. Lazarus S. (2003). Tomatoes and cardiovascular health. Crit. Rev. Food Sci. Nutr. 43 (1), 1–18. 10.1080/10408690390826437 12587984

[B74] Wittenauer J. Mäckle S. Sußmann D. Schweiggert-Weisz U. Carle R. (2015). Inhibitory effects of polyphenols from grape pomace extract on collagenase and elastase activity. Fitoterapia 101, 179–187. 10.1016/j.fitote.2015.01.005 25598188

[B75] Yamamoto K. (2017). Food processing by high hydrostatic pressure. Biosci. Biotechnol. Biochem. 81 (4), 672–679. 10.1080/09168451.2017.1281723 28300504

